# The altered glucose metabolism in tumor and a tumor acidic microenvironment associated with extracellular matrix metalloproteinase inducer and monocarboxylate transporters

**DOI:** 10.18632/oncotarget.8153

**Published:** 2016-03-17

**Authors:** Xiaofeng Li, Xiaozhou Yu, Dong Dai, Xiuyu Song, Wengui Xu

**Affiliations:** ^1^ Department of Molecular Imaging and Nuclear Medicine, Tianjin Medical University Cancer Institute and Hospital, Tianjin, China; ^2^ National Clinical Research Center for Cancer, Tianjin, China; ^3^ Key Laboratory of Cancer Prevention and Therapy, Tianjin, China

**Keywords:** extracellular matrix metalloproteinase inducer, monocarboxylate transporters, glycolysis, tumor acidic microenvironment, p53

## Abstract

Extracellular matrix metalloproteinase inducer, also knowns as cluster of differentiation 147 (CD147) or basigin, is a widely distributed cell surface glycoprotein that is involved in numerous physiological and pathological functions, especially in tumor invasion and metastasis. Monocarboxylate transporters (MCTs) catalyze the proton-linked transport of monocarboxylates such as L-lactate across the plasma membrane to preserve the intracellular pH and maintain cell homeostasis. As a chaperone to some MCT isoforms, CD147 overexpression significantly contributes to the metabolic transformation of tumor. This overexpression is characterized by accelerated aerobic glycolysis and lactate efflux, and it eventually provides the tumor cells with a metabolic advantage and an invasive phenotype in the acidic tumor microenvironment. This review highlights the roles of CD147 and MCTs in tumor cell metabolism and the associated molecular mechanisms. The regulation of CD147 and MCTs may prove to be with a therapeutic potential for tumors through the metabolic modification of the tumor microenvironment.

## INTRODUCTION

A majority of human tumors exhibit significantly higher glucose flux compared with adjacent normal tissues, and the glucose metabolism is characterized by increased aerobic glycolysis in the tumorous tissues [1-4]. This metabolic switch provides tumors with a growth and invasion advantage especially under hypoxic conditions. However, increased dependence on glycolysis results in increased lactic acid production, and the abundant lactic acid has to be exported mainly by two H^+^/lactate symporters (monocarboxylate transporters, MCT1/MCT4) in order to prevent cellular acidosis in the tumor cells [5-10]. On the other hand, increased acid efflux due to altered glucose metabolism results in the chronic exposure of peritumoral normal tissues to an acidic microenvironment that produces toxicity in normal cells. Tumor cells evolve resistance to acid-induced toxicity during tumorigenesis, allowing them to invade the damaged surrounding normal tissues.

It has been well recognized that cluster of differentiation 147 (CD147) is broadly expressed on human tumors and plays a critical role in tumor progression [11-17]. Intriguingly, CD147 has also been shown to associate specifically with cell surface expression and the appropriate location of MCTs as a chaperone in the energy metabolism of tumors, thus contributing to the tumor invasion and tumor metastasis [18-21]. In this review, we seek to describe the roles of CD147 and MCTs in altered glucose metabolism and the consequent acid-mediated invasive phenotype of tumors, and we discuss the underlying molecular mechanisms.

## CD147 IN TUMORS

CD147, also known as HAb18G/CD147 in humans, is a hepatoma-associated antigen cloned by hepatoma monoclonal antibody HAb18 screening from a human hepatocellular carcinoma cDNA library [22, 23]. HAb18G/CD147, which belongs to the immunoglobulin superfamily, contains two extracellular immunoglobulin domains (C and V domains), a hydrophobic transmembrane domain, and an intracellular domain [24, 25]. The corresponding gene is located on chromosome 19p13.3 and contains 1797 bp encoding 269 amino acid residues [26-28]. There are three Sp1 binding sites and two hypoxia-inducible factor (HIF) binding sites in the 5’-flanking and the 3’-flanking region, respectively, of the CD147 gene. [29, 30].

In addition to its familiar matrix metalloproteinase (MMP)-inducing ability, CD147 plays a vital role in neural network formation and development [31, 32], spermatogenesis and fertilization [33, 34], lymphocyte responsiveness [35], rheumatoid arthritis, HIV infection [36, 37], tumor metastasis, and tumor angiogenesis [11]. A study involving CD147−/− mice demonstrated defective activities in MMP production and secretion, spermatogenesis, lymphocyte responsiveness, and neurological functions in the early stages of development; these animals were also infertile due to failure of implantation and fertilization [38]. As a type I integral membrane receptor, CD147 typically associates with many ligands, such as MCTs [18-21], integrins [39], annexin II [40], caveolin-1 [41], cyclophilin A [42], and βig-h3 [43], based on the characteristics of the molecular structure of CD147.

As previously reported, CD147 was highly enriched on the surface of various malignant tumor cells, including cancers of the brain, lung, breast, liver, bladder, and skin [12, 13], triggering the production and release of MMPs in the surrounding stromal fibroblasts and endothelial cells, as well as in the tumor cells themselves, *via* cGMP/NO-sensitive capacitative calcium entry. The MMPs thus contributed to the degradation of the extracellular matrix, which eventually led to tumor invasion and metastasis [14-16]. In addition, it has been found that elevated CD147 expression is significantly correlated with the malignancy of these cancers [11, 17].

Vascular endothelial growth factor (VEGF), which can be upregulated under hypoxic and acidic conditions in tumors, plays a pivotal role in tumor angiogenesis and is crucial for tumor growth, invasion, and metastasis [44]. As indicated previously by Tang et al.[45], modulating CD147 expression and activity *via* recombinant DNA engineering and neutralizing antibodies influenced VEGF production at both the RNA and protein levels in human breast cancer cells in a CD147- and MMP-dependent fashion in cocultures of tumor cells and fibroblasts. Consistently, CD147 regulated VEGF and MMP expression in xenograft tumors and stimulated tumor angiogenic potential and growth rate. Similarly, the knocking down of CD147 using specific siRNA significantly inhibited VEGF expression by malignant melanoma cells, resulting in the suppression of microvessel density in nude mouse xenograft models [17]. These findings strongly support the idea that in addition to being an MPP inducer, tumor-associated CD147 is still an important angiogenesis enhancer that contributes to the tumor angiogenesis mechanism in tumor progression [17, 45, 46]. To explore the underlying signaling pathways used by CD147 to induce VEGF expression, previous studies by Tang et al. have shown that the PI3K-Akt signaling pathway is specifically involved in the regulation of VEGF expression by CD147 in MDA-MB-231 breast cancer cells. The researchers used various blocking and neutralization experiments aimed at PI3K and CD147, and they identified a positive feedback regulatory mechanism of CD147 expression [47, 48]. In addition, Sounni et al. suggested that membrane type 1 MMP induced by CD147 might specifically stimulate VEGF-A production directly *via* the Src tyrosine kinase signaling pathway in human breast carcinoma MCF7 cells [49]. The PI3K-Akt signaling pathway has been well accepted as one of the most important signaling pathways in angiogenesis, as shown previously by a series of evidence [50].

Cell^−^cell or cell^−^matrix anchorage is important for cell viability and proliferation, as loss or alteration of this anchorage could lead to anoikis which is a form of apoptosis [51]. The acquisition of anoikis resistance is a key feature of neoplastic transformation, and it is an important prerequisite for tumor invasion and metastasis. Previous findings have indicated that CD147 expression protects breast cancer cells from anoikis, at least in part, by a mitogen-activated protein kinase-dependent reduction of Bim, which is a proapoptotic BH3-only protein, and that knockdown of CD147 expression by RNA interference sensitized cancer cells to anoikis through the activation of caspase-3 [52]. Ke et al. found that CD147 expression was significantly higher in hepatocellular carcinoma cells (SMMC-7721) resistant to anoikis compared with the parental cells, and that CD147 knockdown by siRNA also remarkably induced cell anoikis, partially *via* inactivation of the PI3K/Akt signaling pathway [53]. In summary, the acquisition of anoikis resistance through upregulation of CD147 may represent a newly recognized mechanism underlying the metastasis of malignant tumor cells. In addition to cell apoptosis and necrosis, autophagy is usually known as another important form of cell death [54], and the role of autophagy in tumors has been a topic of intense discussion [55, 56]. It has been reported that HAb18G/CD147 inhibited the starvation-induced formation of autophagosomes in human SMMC-7721 liver cancer cells in a dose-dependent manner *via* downregulation of autophagy-relating protein ATG6/Beclin1 expression involving the Class I PI3K/AKT pathway [57].

It is known that CD147 is highly expressed on the hepatocellular carcinoma cells (HCCs) and is involved in tumor growth, angiogenesis, invasion, and metastasis as an early pathological diagnosis biomarker and a significantly unfavorable prognostic factor for HCC [16, 58, 59]. In detail, CD147 colocalized and interacted with integrin α3β1 [39] and α6β1 [60] in the invasion and metastasis potential of hepatoma cells *via* the integrin α3β1-mediated focal adhesion kinase (FAK)-paxillin and FAK-PI3K-Ca2^+^ signaling pathways, and the integrin α6β1-mediated PI3K-Ca2^+^ signaling pathways, respectively. The enhancing effects of HAb18G/CD147 on the adhesion, invasion capacities and secretion of MMPs of hepatoma cells (SMMC-7721) were partially blocked by integrin α3β1 and α6β1 antibodies; Wortmannin and LY294002, specific PI3K inhibitors, were able to reverse the attenuating effect of HAb18G/CD147 on the negative regulation of Ca2^+^ entry by PI3K. Further study about the fundamental mechanisms underlying the interaction between CD147 and integrin confirmed that the extracellular membrane-proximal domain of CD147 bonds to the metal ion-dependent adhesion site (MIDAS) motif of integrin β1 to activate the downstream FAK signaling pathway, subsequently functioning in the invasion and metastasis of HCCs [61]. In the same manner, the RGD motif in many extracellular matrix proteins interacted with the MIDAS motif of integrin β1 submit [62]; therefore the RGD motif may have competitively inhibited the CD147^−^integrin β1 interaction and attenuated the malignant properties of tumor cells induced by CD147, which would be a potentially innovative therapeutic strategy for tumors.

In addition to integrin, annexin II, a 36-kDa Ca2^+^ and phospholipid-binding protein, has been characterized as a new interaction protein of CD147 in HCCs to promote the invasion and migration of HCCs *in vitro* as a functional complex in the same signal transduction pathway [40]. However, the expression of annexin II was not affected by the downregulation of CD147, and vice versa [40]. Cytoskeleton rearrangement plays an important role in cell motility. The annexin II^−^CD147 interaction is involved in the cytoskeleton rearrangement of HCCs *via* inhibiting Rho/ROCK signaling pathways and amoeboid movement by CD147 through inhibiting the phosphorylation of annexin II, thus promoting membrane localization of WAVE2 and Rac1 activation, and contributing to the formation of lamellipodia and mesenchymal movement by way of the integrin-FAK-PI3K/PIP3 signaling pathway [63]. Epithelial^−^mesenchymal transition (EMT) is defined as a process in which stationary polarized epithelial cells are converted into motile mesenchyma-like cells with an invasive phenotype and malignant behavior [64], triggered by TGF-β *via* Smad-dependent and non-Smad-dependent signaling pathways [65]. Interestingly, research results from Wu and his group uncovered, for the first time, a novel role of CD147 in mediating EMT. Samples from patients with liver disease indicated that the expression of HAb18G/CD147 was upregulated in TGF-β-induced EMT. A dual-luciferase reporter assay and ChIP further demonstrated that CD147 upregulation was controlled by the PI3K/Akt/GSK3b signaling pathway, and that CD147 was a transcriptional target of Slug [66].

**Figure 1:**
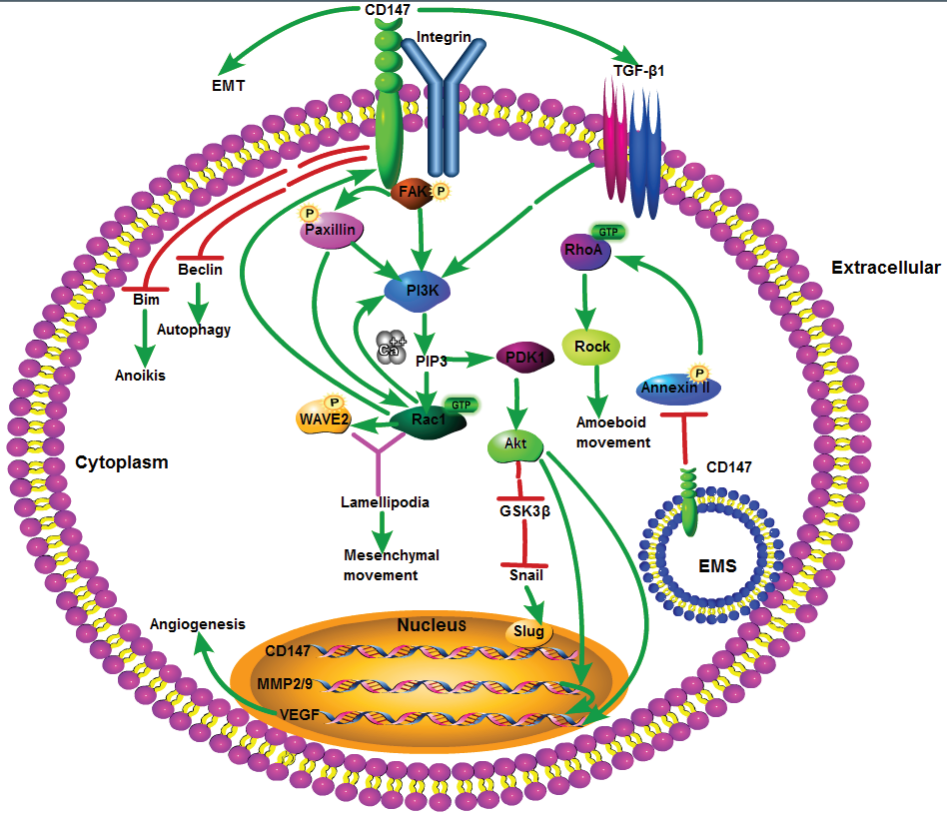
Schematic representation of the non-metabolic molecular mechanism of tumor progression associated with CD147 overexpression First, CD147 colocalizes and interacts with integrin in the invasion and metastasis of tumor cells *via* integrin α3β1-mediated FAK-paxillin and FAK-PI3K-Ca^2+^ signal pathways and integrin α6β1-mediated PI3K-Ca^2+^ signaling pathways, respectively. Second, the annexin II^−^CD147 interaction is involved in rearranging the cytoskeleton *via* inhibiting Rho signaling pathways and amoeboid movement by CD147 through inhibiting annexin II phosphorylation in the EMS, thus promoting membrane localization of WAVE2, Rac1 activation, formation of lamellipodia and mesenchymal movement *via* the integrin-FAK-PI3K/PIP3 signaling pathway. Third, CD147 stimulates tumor angiogenesis by elevating VEGF and MMPs *via* PI3K-Akt signaling pathway. In addition, CD147 is involved in EMT *via* a signaling cascade: TGF-β^−^PI3K/Akt^−^GSK3β^−^Snail^−^Slug^−^CD147. Finally, CD147 expression protects tumor cells from anoikis and starvation-induced autophagy at least in part by reducing Bim and downregulating autophagy-relating gene ATG6/Beclin1, respectively. Akt: also known as protein kinase B (PKB); Bim: Bcl-2 interacting mediator of cell death; EMT: epithelial mesenchymal transition; EMS: endomembrane system; FAK: focal adhesion kinase; GSK-3β: glycogen synthase kinase-3β; MMP: matrix metalloproteinase; PDK1: phosphoinositide dependent protein kinase-1; PI3K: phosphatidylinositol 3-kinase; PIP3: phosphatidylinositol 3,4,5-trisphosphate; Rac1: Ras-related C3 botulinum toxin substrate 1; RhoA: Ras homolog gene family, member A; Rock: Rho-kinase; Snail: zinc-finger transcriptional factor Snail; Slug: zinc-finger transcriptional factor Slug; TGF-β1: transforming growth factor-β1; VEGF: vascular endothelial growth factor; WAVE2: WASP-family verprolin homologous protein 2.

In conclusion, CD147 is a tumor-associated antigen involved in the growth, survival, invasion, angiogenesis, and metastasis of tumors, mainly *via* CD147-mediated MMP production and interaction with various ligands involved in the neoplastic cell behavior. All mentioned non-metabolic molecular mechanisms of tumor progression associated with CD147 overexpression are represented schematically in Figure 1.

## MCTS IN TUMORS

MCTs belong to the solute carrier 16 gene family, which currently contains 14 members [67, 68]. All family members are predicted to have 12 transmembrane helices (TMs) with intracellular C- and N-termini and a large cytosolic loop between TMs 6 and 7 [67]. MCTs catalyze the transport of monocarboxylates such as L-lactate across the plasma membrane. However, only four members of the family (MCTs1^−^4) have actually been confirmed to function as proton-linked MCTs, whereas MCT8 is a thyroid hormone transporter [69] and MCT10, originally known as T-type amino acid transporter 1, is an aromatic amino acid transporter [70]. Transport mediated by MCT8 and MCT10 is not proton linked. The essential metabolic roles of MCT isoforms 1^−^4 in cell homeostasis has been depicted in detail in most normal tissues. Depending on the tissues and the species, MCT1 or MCT2 is used to take up lactic acid for energy metabolism (e.g., oxidation in heart, red muscle, and neurons) or for gluconeogenesis (liver and kidney) [71-74]. MCT4 plays a critical role in lactic acid efflux in most tissues that rely on glycolysis for energy metabolism under normoxic conditions (e.g., white skeletal muscle fibers) [75, 76]. MCT3 expression is confined to the retinal pigment epithelium (RPE) and choroid plexus [77], and it is believed to facilitate the transport of glycolytically derived lactic acid out of the retina.

MCT expression can be regulated at both the transcriptional and post-transcriptional levels [68]. Numerous studies have reported the upregulation of MCT1 in skeletal muscle in response to chronic stimulation or exercise at the transcriptional level through elevated calcium and AMP-activated protein kinase (AMPK) [72]. MCT2 expression may be subject to post-transcriptional control. It has been reported that noradrenaline and both insulin and insulin-like growth factor (IGF)-1 enhance the expression of MCT2 by translational activation mediated by stimulation of the PI3K/Akt/mTOR pathway [78]. However, how such a restricted expression of MCT3 in RPE and the choroid plexus is regulated is not actually known. Among the major regulatory mechanisms identified for MCT4 expression, the upregulation of MCT4 expression in response to hypoxia mediated by HIF-1α is of particular importance [79]. Hypoxia could increase MCT4 mRNA and protein expression, as MCT4 promoter activity is stimulated by hypoxia *via* the presence of four potential hypoxia response elements in the MCT4 promoter [79]. This is consistent with the proposed role of MCT4 in pumping out lactic acid derived from glycolysis across the plasma membrane from cells; elevated MCT4 expression is often observed in tumor cells that rely almost entirely on glycolysis for their energy metabolism [80].

One of the recently recognized hallmarks of cancer is altered glucose metabolism with dependence on glycolysis for energy production [4]. Consequently, large amounts of lactate produced have to be exported to the extracellular milieu; thus, it is not surprising that cancer cells exhibit high levels of MCT expression to maintain this metabolic phenotype [9, 10]. In this context, MCT1 and MCT4 on one hand play a dual role in the maintenance of high glycolytic rates by performing lactate efflux, and on the other hand contribute to the homeostasis of the intracellular pH through the cotransport of protons [81]. However, there is considerable variation in the expression of MCT isoforms in different tumors due to the different metabolic profiles of tumors [82].

In the literature, there is controversy regarding the expression of MCTs in colorectal carcinoma [83, 84] and breast cancer [85, 86]. Regarding tumors of the central nervous system, a recent report indicated that MCT1 and MCT4 are overexpressed in the plasma membrane of glioblastoma cells [87]. There is only one study showing a dramatic increase in the expression of MCT1 and MCT4 from pre-invasive to invasive squamous lesions in the uterine cervix [88]. It has also been reported that MCT1, MCT2, and MCT4 are highly expressed in gastrointestinal stromal tumors and are significantly associated with clinicopathological data [89]. While one study reported an increase of both MCT1 and MCT 4 expression and a positive correlation with prostate cancer progression [90], another report demonstrated a dramatic upregulation of both MCT 2 and MCT4, but a significant decrease in MCT1 expression in prostate cancer cells [91]. Additional studies regarding MCT expression in other tumor types, verification of the reports already published, and further excellent functional studies are needed for a more in-depth elucidation of the importance of MCTs in cancer.

## CD147 AND MCTS IN TUMOR GLYCOLYSIS

One characteristic of the altered metabolism in malignant tumor is the increased glucose uptake [1]. Differential diagnosis between malignant and benign lesions using positron emission tomography imaging is based on this fundamental feature of tumor metabolism [92]. In contrast to the corresponding normal tissues which depend on mitochondrial aerobic respiration for the production of energy in the presence of physiologic oxygen and glycolysis to metabolize glucose during oxygen deprivation [93], tumor tissues mainly use aerobic glycolysis to metabolize glucose even in the presence of sufficient oxygen [2-4]. This metabolic adaption within the malignant tumor was first identified by Otto Warburg in 1956 and called the Warburg effect [94].

As mentioned previously, CD147 interacts with MCTs [18-21], and it has been shown to serve as a chaperone to assist MCT1 and MCT4 in folding, stability, membrane expression, and functionality [19]. Specifically, CD147 regulates the surface expression and function of MCT1, which is generally widely expressed [95]. The expression of MCT4, however, which could be induced under the condition of hypoxia, tended to be restricted to tissues utilizing glycolysis, and increased expression of MCT4 has been reported in several malignant tumors [67, 79, 80]. A hallmark of the altered metabolism in malignant tumors is aerobic glycolysis. Blocking CD147 with a targeted monoclonal antibody or silencing CD147 by siRNA resulted in the inhibition of the proliferation, invasiveness, angiogenesis, and metastatic potential of colon cancer cells and malignant melanoma cells [17, 96, 97]. These results potentially suggest that the protumoral action of CD147 is at least in part due to the interaction with MCT1/MCT4 to promote tumor cell glycolysis *via* increased glucose uptake, lactate release, and the production of adenosine triphosphate (ATP). MCT4, a hypoxia-inducible and tumor-associated lactate/H+ symporter, has been shouwn to confer resistance to the suppression of growth of Ras-transformed fibroblasts (glycolytic tumors) by MCT1/2 inhibition and to reestablish the tumorigenicity [98].

Hypoxia, one of the most pervasive physiological stresses within the tumor microenvironment [99], is largely due to poorly formed tumor vasculature [100]. Significant regions of tumor tissues are separated from the supporting blood vessels by great distances, causing hypoxia, nutrient deficiency, and waste product accumulation [101]. Tumor cells have been shown to undergo fundamental metabolism adaption in order to survive and to display a growth advantage in the tumor microenvironment with limited oxygen and nutrition [2-4]. A series of transcription factors have been reported to be implicated in this process [102, 103], among which, HIF-1α transcription factor [104, 105] plays a pivotal role in this hypoxic adaption, mainly *via* the overexpression and increased activity of several glycolytic proteins, including glucose transporter-1 (GLUT-1), MCT-4, and a variety of glycometabolic enzymes [21, 80]. A genome-wide chromatin immunoprecipitation (ChIP)-on-chip assay identified CD147 as a new hypoxia-responsive molecule essential for the glycolytic switch under hypoxia [106, 107]. Increased expression of CD147 at both the mRNA and protein levels in a time- and dose-dependent manner in the hypoxic microenvironment of epithelial solid tumor has been revealed by immunohistochemical staining [21]. The identification of the key molecules involved in tumor hypoxia adaptation confirmed that CD147 up-regulation was mainly mediated by a combined effect of HIF-1α and specificity protein 1 (Sp1) on the activation of CD147 promoter [21]. Kong et al. also found that promoter hypomethylation up-regulated CD147 expression primarily through increased Sp1 binding and that it was associated with a poor prognosis in human hepatocellular carcinoma [107]. As expected, hypoxia-induced CD147 enhanced glycolysis in both tumor cell lines and a tumor xenograft model, partially through interaction with MCT-1 and MCT-4 [21]. In summary, a body of evidence uncovered a novel mechanism of hypoxia adaptation mediated by the interaction between CD147 and MCTs to promote glycolysis in tumor progression.

Recently, the enthusiasm to study the importance of metabolism in cancer has resulted in some attention-grabbing discoveries that revealed additional functions of well-known oncogene and tumor suppressor gene-encoded proteins [108]. Over the past 15 years, increasing evidence has proven that oncogene Myc, PI3Ks/AKT/mTOR pathways, along with HIF, can stimulate the transcription of a number of genes whose products are significantly involved in glycolysis pathways [109, 110]. Similarly, p53, which is mostly known for its tumor suppressor properties, is also able to control the metabolic switch in cancer directly through a series of mechanisms that decelerate glycolysis and help to maintain aerobic respiration [111, 112]. The p53 protein has been shouwn to inhibit the expression of GLUT 1 and GLUT4 transporters, which are important for glucose uptake [113]. More intriguingly, p53 is also able to inhibit the activities of PI3K/AKT/mTOR signaling pathways by regulating the transcription of four important genes, including insulin-like growth factor 1-binding protein-3 (IGF-1BP-3), tuberous sclerosis protein 2 (TSC-2), phosphatase and tensin homolog (PTEN) and the beta subunit of AMP-activated protein kinase (AMPK) which all negatively regulate AKT and mTOR activities [114].

**Figure 2:**
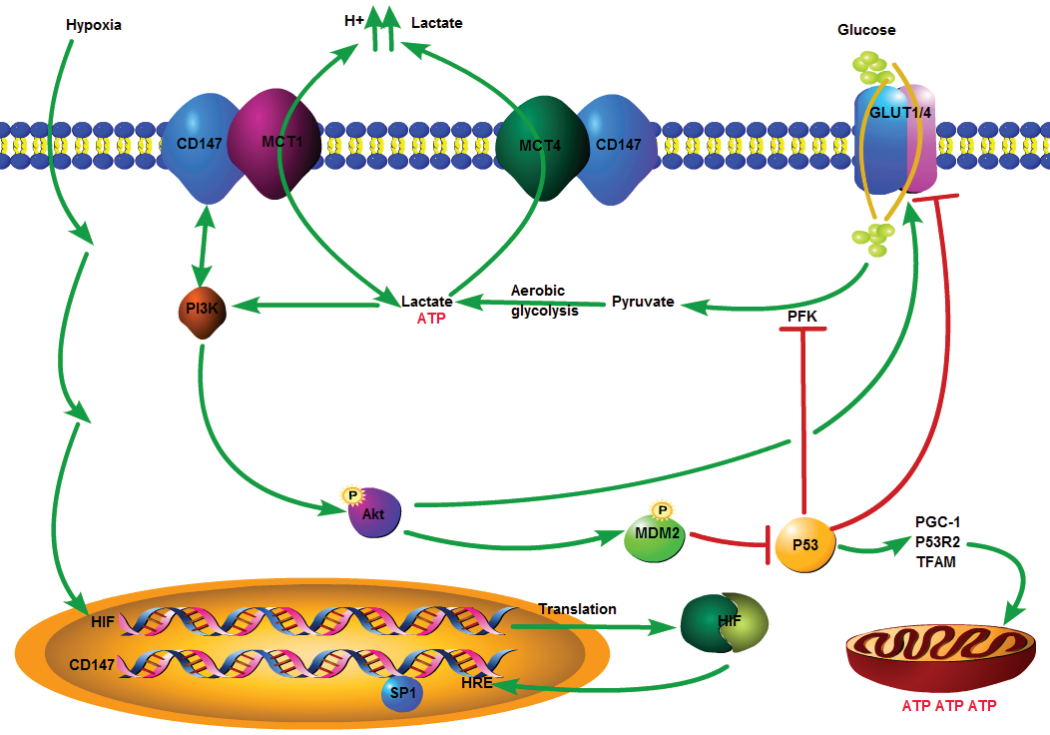
Schematic depicting the regulation of altered glucose metabolism by CD147 in a tumor Hypoxia in the tumor microenvironment induces the upregulation of CD147 expression by a combined effect of transcription factor HIF-1 on HRE of CD147 and SP1 on the activation of CD147 promotor. The overexpression of CD147 promotes aerobic glycolysis mediated by the activation of the PI3K/Akt/MDM2 pathway, subsequently promotion of p53 degradation and inhibiting the downregulation of GLUT1/4 gene expression and suppression of PFK by p53 in the glycolytic metabolism and repressing mitochondrial oxidative respiration *via* downregulation of PGC1a, TFAM, and p53R2 in a p53-dependent manner at the same time. The glycolytic phenotype of tumor leads to increased production of lactic acid, which has to be exported across the plasma membrane in order to prevent cell death, due to cellular acidosis. Lactate is pumped out from cells mainly through two H+/lactate co-transporters, MCT1 and MCT4, to maintain homeostasis in the intracellular pH of tumors (MCT1 is bidirectional). CD147 serves as a chaperone to assist in the surface expression, folding, stability, appropriate location and functionality of MCT1 and MCT4. ATP: adenosine triphosphate; Akt: also known as protein kinase B (PKB); GLUT1/4: glucose transporter 1/4; HIF: hypoxia-inducible factor; HRE: hypoxia response element; MCT1/4: monocarboxylate transporter 1/4; MDM2: mouse double minute 2 homolog; PFK: phosphofructokinase; PGC-1: peroxisome proliferators-activated receptor-γ coactivator-1; PI3K: phosphatidylinositol 3-kinase; P53: protein 53; P53R2: p53-inducible ribonu cleotidereductase small submit 2; SP1: specificity protein 1 transcription factor; TFAM: mitochondrial transcription factor A.

As mentioned above, several intracellular signaling mediators have been identified in the metabolic regulation of tumor cells. However, relevant reports regarding the involvement of some molecules on the cell surface in the reprogramming process of glucose metabolism are rare and ambiguous. Several recent investigations have determined the involvement of CD147 in tumor glycolytic metabolism through the gain/loss-of-function studies [21, 98]. A study by Huang and his colleagues [115] demonstrated that CD147 contributed significantly to the altered glucose metabolism in HCCs through a p53-dependent way (Figure 2). First, upregulation of CD147 promoted glycolysis mediated by the p53-dependent upregulation of GLUT1 and activation of liver type phosphofructokinase (PFKL) in HCC lines. However, the increased expression of CD147 inhibited mitochondrial oxidative resipiration mediated by the p53-dependent downregulation of PGC1a, TFAM, and p53R2 at the same time. Second, CD147 triggered the activation of the PI3K/Akt/MDM2 pathway and the subsequent promotion of p53 degradation, thus accelerating the lactate export through MCT1 in HCCs [115]. Regulation of the altered glucose metabolism by CD147 and MCT1/MCT4 in tumor is depicted schematically in Figure 2.

There are currently several drugs in clinical trials or under development that are based on specifically targeting the aberrant metabolism of tumors [109]. The therapeutic strategies include indirect targets (signaling pathways that are involved in altered glucose metabolism in the tumor) and direct targets consisting of the metabolic enzymes themselves (targeting nucleotide biosynthesis, glycolysis, amino acid metabolism and lipid metabolism). A study by Huang [114] demonstrated that the *in vitro* and *in vivo* proliferation of HCCs cells was suppressed by knockout or blocking CD147 and/or MCT1, which resulted in the down-regulation of glucose metabolism, suggesting that CD147 is a promising therapeutic target in HCCs by reprogramming the glucose metabolism. Actually, ^131^I-labeled radioimmunologic monoclonal antibody against CD147 agent (generic name: [^131^I] metuximab injection; brand name: Licartin), was previously developed and approved by Sino FDA for clinical application [116, 117]. An increasing body of evidence has revealed a previously unrecognized metabolic mechanism of application of CD147 in cancer diagnosis and therapeutic intervention.

## TUMOR ACIDIC MICROENVIRONMENT AND TUMOR PROGRESSION

It is well accepted that tumor microenvironments play a significant role in modulating tumor development and progression [118]. Hypoxia and lactic acidosis are two important determinants in this environment, and tumor cells adapt their metabolism to respond to these unauspicious conditions [80]. As mentioned above, increased glucose uptake and elevated aerobic glycolysis, which are induced maily by HIF-1 under hypoxia, confer tumor cells a remarkable growth advantage. As such, the increased acid production due to the altered glucose metabolism subjects the peritumoral normal tissue to chronic exposure to an acidic microenvironment. This increased production of glycolytically derived acid is toxic to the surrounding normal cells due to the caspase-mediated activation of the p53-dependent apoptosis of normal cells that express wild-type p53 activity [119, 120]. Tumor cells have evolved a resistance to acid-mediated toxicity during carcinogenesis, permitting them to invade the damaged adjacent normal tissues. The constitutive upregulation of H^+^ transporters or mutations in p53 and/or the downstream effectors in tumor cells might partially contribute to the tolerance of tumor cells to acidosis of the microenvironment [6, 121, 122]. We hypothesize that the glycolytic phenotype first emerged as a survival mechanism to adapt to the hypoxic microenvironment, and that the increased acid production from the upregulated glycolysis led to acid-mediated tumor invasion. In addition to evoking different sensitivities to the acidic microenvironment in normal cell competitors and tumor cells, extracellular acidosis also promotes angiogenesis through the enhanced release of VEGF [123, 124] and indirectly accelerated extracellular matrix degradation by inducing adjacent fibroblast and macrophage cells to release proteolytic enzymes such as cathepsin B [125] or by increased lysosomal recycling [126].

Tumor-infiltrating lymphocytes, an important part of the tumor surveillance system, play an important role in the anti-tumor immunity [127]. However, the spontaneous clearance of established tumor lesions by endogenous immune mechanisms is rare. Increasing evidences has attributed this phenomenon to a functional impairment of effector T cells in the tumor microenvironment [128]. Activated T cells also rely on elevated glycolysis and efficient secretion of lactic acid due to a higher energy demand during proliferation and cytokine production [129, 130]. The transcriptional and translational expression of MCT1, 2 and 4 has already been reported in lymphocytes [131]. However, a disadvantageous lactic acid gradient between the extracellular milieu and the cytoplasm due to an accumulation of lactic acid in the tumor environment suppresses the proliferation and cytokine production of effector T cells *via* blockade of lactate efflux, thereby disturbing T-cell metabolism [130]. Consistent with those findings, extracellular lactic acidosis derived from tumors has also been shown to modulate the antigen-presenting capability of human monocyte-derived dendritic cells (DCs) to differentiate into tumor-associated DCs in a three-dimensional tumor model within multicellular tumor spheroids [132]. As we know, elevated expression of CD147 has been observed on activated lymphocytes and is involved in the immunological synapse formation [133]. Furthermore, CD147 in regulatory T cells has been able to identify FoxP3^+^CD45RO^+^CTLA4^+^ activated human regulatory T cells from resting regulatory T cells [134, 135]. In other words, CD147 is engaged in both immune response and immune suppression.

## THERAPEUTIC POTENTIAL OF CD147 AND MCTS IN TUMORS

It has been suggested that CD147 detection is a useful test for the pathological diagnosis of early hepatocellular carcinoma in needle biopsy samples [58]. Multivariate analysis has revealed that the expression of CD147 is an independent prognostic indicator for patients with HCC and non-small cell lung cancer [58, 136]. In addition, low CD147 expression has also been suggested as a significantly favorable prognostic factor in gastric carcinoma [137], glioblastoma [138], endometrial cancer [139], and hypopharyngeal squamous cell carcinoma [140]. In patients with urothelial carcinoma of the bladder (UCB), univariate analysis revealed that high MCT1 and CD147 expressions were correlated with poor overall survival, whereas high MCT4 expression was associated with poor recurrence-free survival. Multivariate analysis has also indicated that high MCT1 and MCT4 expression can be independent prognostic markers for poor overall survival and poor recurrence-free survival, respectively [141].

The clinical application of CD147 and MCTs has been suggested not only as a potential diagnostic and prognostic marker in tumors [142], but also as a potential therapeutic target. It has been reported that anti-CD147 monoclonal antibody and ^131^I-labeled HAb18 F(ab’)_2_ metuximab monoclonal antibody injection (Brand name: Licartin), as a targeted radioimmunotherapy for HCC patients significantly decrease the secretion of MMPs and the invasive potential of HCC cells, which could be used to effectively prevent the recurrence and metastasis of HCC after hepatectomy and liver transplantation [16, 143]. A combination therapy of percutaneous radiofrequency ablation (RFA) and ^131^I-labeled metuximab treatment showed a greater anti-recurrence benefit than RFA alone [144]. Chimeric CD147 antibody as referred to CNTO 3899 was evaluated as a potential treatment for head-and-neck squamous cell carcinoma by means of inhibition of cytokines, MMPs and VEGF [145, 146]. From the perspective of tumor metabolism, CD147 modulation disrupted its interaction with MCTs and rendered the tumor cells vulnerable to energy deficiency as they are dependent on aerobic glycolysis, which is regulated through the cooperation of MCTs and CD147, for their energy supply [147]. However, CD147 is broadly expressed on hematopoietic cells, and which play a critical role in a series of physiological activities, such as lymphocyte activation, so the application and consequence of anti-CD147 should be evaluated comprehensively in more details.

MCTs play a vital role in monocarboxylate transport and pH homeostasis [7]. Because MCT1 is bidirectional, its inhibition not only causes a decrease in intracellular pH and eventually leads to cell death, but also resultes in a more acidic extracellular environment, which is usually associated with a more aggressive behavior of tumor [148-150]. The potential application of MCT1 inhibition in cancer treatment might be explained by a hypothesis referring to a metabolic symbiont model between hypoxic and aerobic cells within the tumor microenvironment [151]. Briefly, in the presence of MCT1, the aerobic tumor cells adjacent to the tumor vessels supply consume lactate to provide a survival advantage to the hypoxic tumor cells far away from the tumor vessels. However, in the absence on MCT1, the aerobic cells have to take up glucose for energy metabolism, due to the unavailability of lactate consumption resulting in susceptibility to starvation of the hypoxic cells. It had been demonstrated that the silence of MCT1 in conjunction with MCT2 could inhibit tumor growth and incurred tumor cell apoptosis and necrosis [152]. On the other hand, inhibition of MCT4 *via* siRNA has been found to remarkably suppress the transwell migration of MDA-MB-231 cells by as much as 85% [153].

Intervention in MCT expression presents clinical application potential, especially in cancer treatment, through cell migration suppression, cell death induction *via* intracellular acidification and hypoxic cell starvation [154, 155]. However, there are no relevant products currently in clinical trials. When considering MCTs as targets for therapy, it is crucial to keep in mind that inhibiting MCTs would trigger deleterious systemic side effects, as it has definitively been found that they are closely associated with a broad range of cell physiological activities. Specifically, systemic delivery of an MCT1 inhibitor could bring disaster to almost every organ of the body, with the most drastic effects occurring on cardiac and skeletal muscles [79]. Therefore, local targeted delivery is required as a first step toward a potential clinical application. Recent evidences from a large cohort of human prostate tissues of different grades supportes MCTs as potential targets in prostate cancer [8]. In the end, because some MCT sub-types rely on CD147 as their chaperone for expression and appropriate location, CD147 inhibition would also contribute to cell apoptosis by disturbing lactate influx/efflux and indirectly lead to a drop in intracellular pH [156].

## CONCLUSIONS

Increased aerobic glycolysis has been well accepted as a remarkable hallmarker of tumors. Increased glucose uptake and elevated aerobic glycolysis provide tumor cells with a remarkable growth advantage, and subsequently, increased acid production that resultes in an acidic tumor microenvironment. Extracellular acidosis leads to tumor progression through several complicated positive feedback pathways, due to different sensitivities to the acidic microenvironment between the normal cell competitors and tumor cells. In view of the interaction between CD147 and MCTs, a previously unrecognized metabolic mechanism of application of CD147 and MCTs in cancer diagnosis and therapeutic intervention has attracted much attention. CD147 and MCTs are increasingly being recognized as potential therapeutic targets in tumors, due to their importance in tumor metabolic switch and eventual tumor progression.
